# Bimanual non-congruent actions in motor neglect syndrome: a combined behavioral/fMRI study

**DOI:** 10.3389/fnhum.2015.00541

**Published:** 2015-10-06

**Authors:** F. Garbarini, L. Turella, M. Rabuffetti, A. Cantagallo, A. Piedimonte, E. Fainardi, A. Berti, L. Fadiga

**Affiliations:** ^1^SAMBA (SpAtial, Motor and Bodily Awareness) Research Group, Department of Psychology, University of TurinTurin, Italy; ^2^Istituto Italiano di Tecnologia (IIT)Genova, Italy; ^3^Center for Mind/Brain (CIMeC), University of TrentoTrento, Italy; ^4^Biomedical Technology Department, IRCCS Don Carlo Gnocchi FoundationMilano, Italy; ^5^BrainCarePadova, Italy; ^6^Department of Neuroradiology Unit, Neuroscience and Rehabilitation, Azienda Ospedaliera UniversitariaFerrara, Italy; ^7^Section of Human Physiology, University of FerraraFerrara, Italy

**Keywords:** motor neglect, fMRI, bimanual actions, bimanual coupling effect, supplementary and pre-supplementary motor area (pre-SMA; SMA)

## Abstract

In Motor Neglect (MN) syndrome, a specific impairment in non-congruent bimanual movements has been described. In the present case-control study, we investigated the neuro-functional correlates of this behavioral deficit. Two right-brain-damaged (RBD) patients, one with (MN+) and one without (MN−) MN, were evaluated by means of functional Magnetic Resonance Imaging (fMRI) in a bimanual Circles-Lines (CL) paradigm. Patients were requested to perform right-hand movements (lines-drawing) and, simultaneously, congruent (lines-drawing) or non-congruent (circles-drawing) left-hand movements. In the behavioral task, MN− patient showed a bimanual-coupling-effect, while MN+ patient did not. The fMRI study showed that in MN−, a fronto-parietal network, mainly involving the pre-supplementary motor area (pre-SMA) and the posterior parietal cortex (PPC), was significantly more active in non-congruent than in congruent conditions, as previously shown in healthy subjects. On the contrary, MN+ patient showed an opposite pattern of activation both in pre-SMA and in PPC. Within this fronto-parietal network, the pre-SMA is supposed to exert an inhibitory influence on the default coupling of homologous muscles, thus allowing the execution of non-congruent movements. In MN syndrome, the described abnormal pre-SMA activity supports the hypothesis that a failure to inhibit ipsilesional motor programs might determine a specific impairment of non-congruent movements.

## Introduction

Motor Neglect (MN) is a neuropsychological syndrome, which occurs as a result of stroke and is characterized by the underutilization of the contralesional limbs, in presence of normal strength, reflexes and sensibility and thus preserved potential for actual movement on the affected side. MN has been described as a “pseudo-hemiplegia” and is often interpreted as the consequence of damage to intentional motor circuits (Laplane and Degos, 1983; Gold et al., 1994; Coulthard et al., 2008; Garbarini et al., 2012, 2013c; Migliaccio et al., 2014). MN, especially in its pure form (without motor deficits), is a rare disorder—Laplane and Degos (1983) collected 20 patients over more than 10 years—and its frequency depends on the phase of the illness. According to some studies, signs of MN occurred in 12–33% of acute stroke patients (e.g., Buxbaum et al., 2004; Siekierka-Kleiser et al., 2006), but the frequency decreased to 8% in chronic patients (e.g., Buxbaum et al., 2004). In one study (Classen et al., 1997), 10 out of 16 patients with MN improved during the first 2 weeks (for a review, see Saevarsson, 2013; see also Migliaccio et al., 2014). Crucial to the present study, when MN patients are asked to perform bimanual movements, they only perform ipsilesional hand movements, even though they are actually capable of moving the contralesional hand. Recently, a behavioral dissociation has been found in MN patients, showing that the underutilization of the affected hand is greater when non-congruent (e.g., to bend one arm while extending the other; to open a bottle…) with respect to congruent (e.g., to clap the hand; to lift up a tray with both hands…) bimanual movements are required (Garbarini et al., 2013c). In the present case-control study, we investigated the neuro-functional correlates of this behavioral dissociation.

We tested two right-brain-damaged (RBD) patients with preserved upper limbs functionality, one with a pure form of MN (MN+) and the other one without MN (MN−), by using a functional Magnetic Resonance Imaging (fMRI) bimanual paradigm (see Section “Materials and Methods”). We took advantage from a Circles-Lines (CL) task (Franz et al., 1991) in which, when people simultaneously draw lines with one hand and circles with the other hand, both trajectories tend to assume an oval shape, showing that hands motor programs interfere (bimanual coupling effect). It has been proposed that such motor constraints are tightly linked to motor intention and planning, rather than to movement execution. In healthy subjects, it has been demonstrated that the interference effect can be modulated by manipulating not the afferent sources of information, but the efferent level of movement planning and organization (Swinnen et al., 2003; Ridderikhoff et al., 2005; Spencer et al., 2005; Dounskaia et al., 2010; de Boer et al., 2013; Garbarini et al., 2015). Accordingly, in pathological conditions, where motor execution is damaged but motor intention is spared, bimanual coupling effects can be observed even in the absence of actual movements of one hand. As suggested by Garbarini et al. (2012), spatial coupling effects are present in RBD patients affected by contralateral (left) hemiplegia and anosognosia for hemiplegia (for temporal coupling effects in anosognosic patients see Pia et al., 2013; see also Garbarini and Pia, 2013). These patients claimed to move both hands when asked to draw lines with their right (intact) hand and circles with their left (paralyzed) hand. Although no movement of the left hand occurred, lines drawn with the right hand showed significant “ovalizations”. Using the same CL paradigm, similar results were also found in amputees with illusory movements of the phantom limb (Franz and Ramachandran, 1998). Using a modified version of the CL task, coupling effects were also found in hemiplegic patients affected by a monothematic delusion of body-ownership, who identified the examiner’s hand drawing circles as belonging to themselves (Garbarini et al., 2013b). In all these pathological conditions, where motor execution is damaged but motor intention is spared, actual movement execution seems unnecessary for bimanual coupling to occur: motor intention and programming are sufficient to trigger the interference effects. On the contrary, when motor execution is spared but motor intention is damaged, as in patients affected by MN, no bimanual constraints were found (Garbarini et al., 2012). The MN cases provide an interesting contrast to the AHP cases. The former are non-plegic but apparently lacking intention/planning, whereas the latter are plegic but still maintain intentions/plans for the affected hand.

According to these behavioral data in brain-damaged patients, previous neuroimaging data in healthy subjects, performing the CL task within the magnetic resonance (MR) scanner, showed the activity of brain circuits related to the intentional and predictive operation generating bimanual coupling (Garbarini et al., 2013a). These results support the role of a prefrontal-parietal network, mainly involving the pre-supplementary motor area (pre-SMA) and the posterior parietal cortex (PPC), that was significantly more active in non-congruent (CL) than in Congruent (Lines-Lines, LL) bimanual conditions.

Based on the above mentioned studies, we expected that MN+ patient, with respect to MN− patient, should show: (a) a worse behavioral performance in non-congruent (CL) than in congruent (LL) conditions; (b) a reduced activity when performing non-congruent conditions in pre-SMA and PPC. Overall, the expected results can reveal the neuro-functional correlates of the behavioral dissociation between congruent and non-congruent movements. More in general, they can represent the first neuro-functional investigation of the MN, shedding light on key areas of the neural network involved in this syndrome.

## Materials and Methods

### Participants

**Figure 1:**
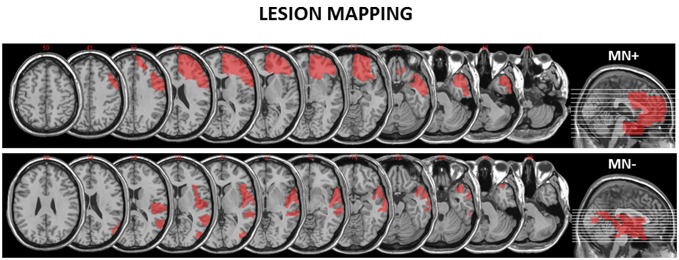
**Reconstruction of the lesions of the motor neglect (MN)+ and MN− patients.** MricroN software was adopted to draw a mask on the patients’ lesions to identify with more precisions the boundaries of brain damage. (http://www.cabiatl.com/mricro/mricron/index.html) **MN+ patient** has a right fronto-temporal cortico-subcortical lesion (lesion extension: 152.34 cm^3^) involving inferior, middle and superior orbital cortex, inferior, middle and superior frontal gyrus, frontal operculum, precentral gyrus, inferior, middle and superior temporal lobe, rostral cingulum bundle. **MN− patient** has a right occipito-temporo-parietal cortical lesion (lesion extension: 106.62 cm^3^) involving middle occipital gyrus, middle temporal gyrus, superior temporal gyrus, rolandic operculum and insula.

We recruited two RBD patients: one MN+ (male; 68 years old) and one MN− (male; 70 years old). The lesion extension of these patients was mapped and measured on the anatomical T1 by using MricroN software^1^ (see Figure 1).

For the present study, we only selected stable patients (in the chronic phase of the illness) able to successfully perform functional task within the MR scanner. Exclusion criteria were: (1) previous neurological or psychiatric history; (2) severe general cognitive impairment; and (3) upper-limb motor deficits. Patients were classified as having or not MN based on clinical considerations, according to the following criteria: (i) spontaneous underutilization of the contralesional upper limb and hand during daily activities; and (ii) contrast between spontaneous underutilization of the left arm and hand, vs. normal movement and strength when the examiner actively encouraged the patient to use the arm. Both MN+ and MN− patients were also assessed using the following tests: general cognitive test (Mini-Mental State Examination—MMSE, Measso et al., 1993; cut off ≥24/30); tests for extrapersonal neglect (Bells Test, Gauthier et al., 1989; cut off omissions *L–R* < 3) and for personal neglect (Fluff Test, Cocchini et al., 2010; cut off omissions *L* ≤ 2); assessment of hemiplegia and hemianesthesia (Pia et al., 2014; scores 0–3, 0 = no deficit; 3 = severe deficit). No deficits were reported in both patients at the time of testing, 1 year after stroke. As reported in the case history, both MN+ and MN− patients showed personal and extrapersonal neglect in the sub-acute phase, within 3 months after stroke. Neurological/neuropsychological assessment is summarized in Table 1.

**Table 1:** **Demographic characteristics, neurological/neuropsychological assessment**.

Patient	MN+	MN−
Age (years)	68	70
Gender	M	M
Education (years)	10	13
Onset (years)	1	1
General cognitive impairement	No	No
Motor-sensory defects	No	No
Personal-extrapersonal neglect	No	No

The patients’ motor performance during both congruent (LL) and non-congruent (CL) bimanual movements within the MRI scanner was evaluated with a score ranging from 0 to 2 (Garbarini et al., 2013c). Each bimanual block (for a total of 12 blocks) was evaluated and the mean score was reported. At the end of the fMRI acquisition, we also asked the patients a self-evaluation, using the same score, of both congruent and non-congruent movements. The examiner’s score and patients’ score are reported in Table 2.

**Table 2:** **Clinical evaluation of the patients’ motor performance during the task**.

**Examiner’s evaluation**
Patient	MN+	MN−
Lines-lines	2	2
Circles-lines	1	2
Patient’s self-evaluation
Patient	MN+	MN−
Lines-lines	2	2
Circles-lines	2	2

*In both the examiner’s evaluation and the patient’s self-evaluation the scores were ranked from 0 to 2: 0 = left hand movements were not performed; 1 = left hand movements were performed but not at the same time of the right hand movements; 2 = left and right hand movements were simultaneously performed. Note that, MN+ patient showed a specific impairment in simultaneously executing non-congruent (CL) movements (i.e., during LL conditions, left and right hand movements were simultaneously performed, score = 2; during CL conditions, left hand movements were performed asynchronously with respect to right hand ones, score = 1). However, a discrepancy emerged between this examiner’s evaluation and the patient’s self-evaluation, where the patient gave the higher score (2) also for the impaired performance at the CL task*.

Both patients gave their informed consent and the protocol was approved by the Ethical Committee “Comitato Etico Unico della Provincia di Ferrara” (Italy).

### Experimental Procedure

Patients were required to perform the “CL” task (Garbarini et al., 2012, 2013c), within a MR scanner while data regarding their brain activity was collected. The CL task involved drawing on a dual panel fMRI compatible tablet (Tam et al., 2012; Garbarini et al., 2013a), using one or both hands, in response to visually administered commitments, A head coil-mounted display system (IFIS-SA, Invivo Corporation, Gainesville, FL) was used to present visual stimuli via E-Prime software (Psychology Software Tools, Inc., Pittsburgh, PA), which also ensured synchronization with the MR scanner and the behavioral data collection. In addition, two of the authors (FG and AP) verified the correct execution of the tasks in the control room.

### Experimental Task and Paradigm

The “CL” task (Garbarini et al., 2012, 2013a), adopted in the present study, consisted in the execution of different unimanual and bimanual motor tasks. The adopted experimental conditions required the patients to perform the following movements:

1. Drawing lines with the right hand (condition L),
2. Drawing circles with the left hand (condition C),
3. Drawing lines with each hand (condition LL),
4. Drawing circles with the left hand and lines with the right hand (condition CL).

This set of behavioral tasks was designed to explore modulations in motor performance of the right (dominant) hand. The behavioral analysis thus enabled quantitative analysis of the interference effect of the controlesional left hand circles drawing, on the lines executed with the ipsilesional right hand.

**Figure 2:**
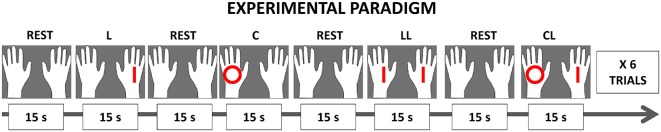
**Experimental paradigm.** The timeline of the study was an alternation of experimental blocks and of rest blocks with the same duration (15 s). A total of 24 experimental blocks (6 repetitions for each of the 4 experimental conditions) was presented to each patient. The study started and ended with a longer rest period (30 s). During the experimental condition blocks, patients had to perform hand movements following the visual cues appearing on the screen within two white hands. Whereas during the rest blocks, patients had to attend the picture depicting two white hands. The possible combination of the observed stimuli matched the experimental conditions: unimanual Lines with the right hand (L); unimanual Circles with the left hand (C); bimanual congruent Lines-Lines (LL), simultaneously with both hands (LL); bimanual non-congruent Circles-Lines (CL), simultaneously with the right hand drawing Lines and the left hand drawing Circles (CL).

The timeline of the study comprised an initial rest of 30 s followed by an alternation of experimental blocks of 15 s duration followed by rest blocks of the same duration. A pseudo-random sequence of experimental blocks was presented to the patients, comprising a total of 24 experimental blocks (6 repetitions for each of the 4 experimental conditions). A final 30 s rest block was presented after all the experimental conditions. During the experimental blocks, the patients had to perform hand movements according to the information (either lines or circles) shown on the head-mounted display (see Figure 2 for a graphical representation of the paradigm).

### Behavioral Data Collection and Analysis

**Figure 3:**
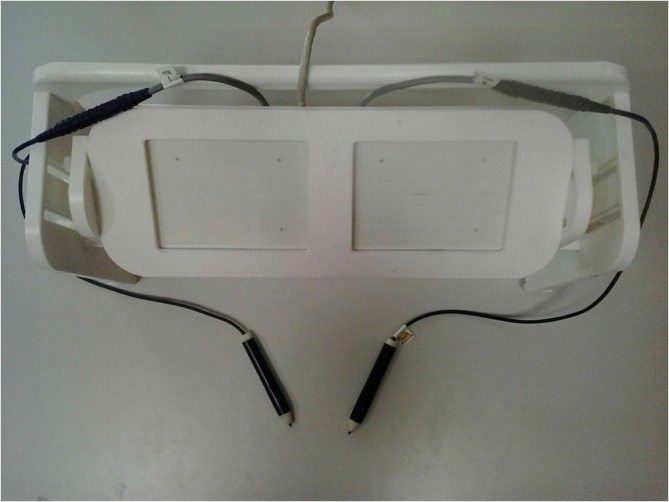
**Dual panel functional Magnetic Resonance Imaging (fMRI)-compatible tablet**.

Behavioral data were collected using a dual panel fMRI-compatible tablet, a modified version of the one used by Tam et al. (2012). This version incorporated two separate panels and two styli allowing the simultaneous collection of data from the two hands (see Figure 3). Behavioral motor performance was recorded from each panel separately by a distinct computer positioned outside the scanner room. Before starting the fMRI study, the patients extensively practiced the task in order to be able to accomplish it smoothly within the scanner.

An Ovalization Index (OI) was defined to quantify the occurrence of lateral deviation when continuously drawing a straight vertical line. The strength of any bimanual coupling/interference effect was signaled by an increased OI value in the Non-congruent condition compared to the Congruent condition.

OI value was defined as the standard deviation of the right-hand trajectories in relation to an absolute vertical line (a detailed description of the algorithm involved in calculating the OI in Garbarini et al., 2012). Briefly, OI index ranges between a value of zero for straight trajectories without any sign of ovalization and a value of 100 for circular trajectories. As a consequence, the value of the OI allows quantifying the bimanual coupling effect for each performed movement by comparing the bimanual movement of each hand with its unimanual equivalent. The amount of interference of the left hand in executing circles on the right hand executing lines is shown as an increase of the OI (bimanual coupling/interference effect). Furthermore, the average drawing frequency was computed for each block as the number of drawing cycles per second, or, alternatively, the inverse of the average cycle duration (in Hz).

### Functional Data Acquisition and Analysis

MR images were acquired on a 1.5T MR scanner (Phillips Achieva). Functional images were collected, while patients were performing the “CL” task, with an EPI T2*-weighted sequence throughout the whole brain (TR = 2500 ms, TE = 50 ms, field of view 230 × 230 mm, in-plane resolution 3.59 × 3.59 mm, slice thickness = 4 mm, 30 slices). A total of 312 images were collected during one functional run. A high quality T1-weighted image (1 mm isotropic voxels) was also acquired to define the lesion extent as shown in Figure 1.

Analysis of fMRI data was carried out by using Statistical Parametric Mapping software^2^. Functional data were realigned using a two-step procedure implemented in SPM5. Data were registered to the first functional volume of the series and then to the mean image. Normalization of the T1-weighted image was performed on the MNI template provided within SPM by using the unified segmentation approach (Ashburner and Friston, 2005) and by applying a masking procedure excluding the part of the brain affected by the lesion. This type of analysis has been demonstrated to strongly improve the normalization procedure in patients with brain lesion (Crinion et al., 2007; Andersen et al., 2010). The resulting normalization parameters were applied to the T1 and to the functional images (resampling the voxels at 2 × 2 × 2 mm). Functional data were spatially smoothed using 8 mm FWHM Gaussian kernel. A high-pass temporal filter (cut-off 128 s) was also applied to the time series. Whole-brain analysis was performed by applying the General Linear Model (GLM) for analysis of fMRI time series. Regressors were defined based on the timing of presentation for each of the conditions and were modeled using a box-car function convolved with the hemodynamic response function (HRF) with duration equal to the experimental block. Predictors of no interest were modeled to account for residual effects of the movements measured during the realignment procedure.

Contrasts of interest were obtained by entering the corresponding contrast vector in the design matrix. The threshold for the presented data was set at a *p* < 0.001 uncorrected for multiple comparisons and reporting only clusters comprising at least 10 voxels. In order to test the role of pre-SMA and PPC in bimanual coupling, we performed a ROI analysis on pre-SMA, left and right PPC. The coordinates for these regions were obtained from a recent study on healthy participants performing the same task (Garbarini et al., 2013a) and transformed from TAL to MNI space adopting the tal2mni function^3^. Beta values were extracted from spherical ROIs (radius 9 mm) centered on the coordinates in MNI space.

### Single-Subject Analyses

In order to analyze behavioral (OI values and drawing frequency) and neuroimaging (beta extracted from the ROI) data, recording from MN+ and MN− patient during the fMRI sessions, we used two different approaches in single-subject analysis: (a) Crawford’s test (Crawford and Garthwaite, 2005) designed to test whether the discrepancy between two tasks (LL; CL) observed for each patient (MN+; MN−) is significantly different from the discrepancies in a control sample; and (b) Crawford’s test (Crawford et al., 2010) designed to test the difference between two single cases (MN+ vs. MN−) by referring to a control sample. For both these methods, which need to refer to normative data from healthy population, we used behavioral and neuroimaging data from healthy participants (*n* = 12) tested in Garbarini et al. (2013a).

## Results

### Behavioral Results

**Figure 4:**
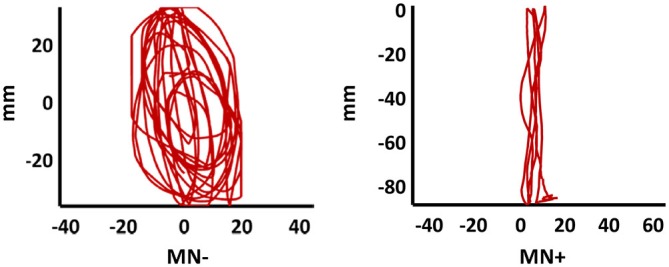
**Patients’ drawing in CL condition.** Examples of patients’ right hand trajectory in bimanual CL condition. Note the evident ovalization for MN− but not for MN+.

In order to quantify the interference (coupling) effects between the two hands motor programming, we analyzed the OI of the different experimental conditions. The bimanual coupling effect should cause, for the right hand always performing lines, the OI value to increase in the non-congruent CL condition (where the left hand performs circles) with respect to the congruent LL condition (where the left hand performs lines). As shown in Figure 4, the MN− patient’s right hand trajectories in CL condition revealed a clear ovalization, while the MN+ patient’s trajectories did not.

**Figure 5:**
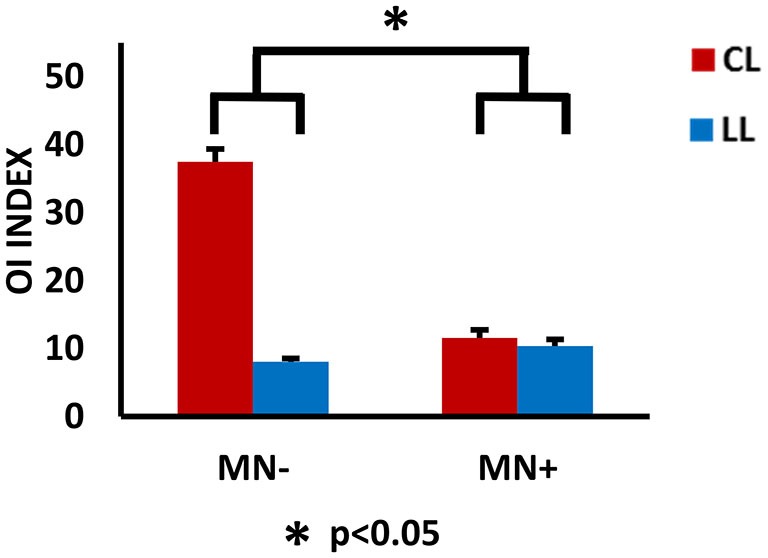
**Behavioral results.** Results of behavioral analysis, with the ovalization index (OI) value for the right hand as dependent variable and CL and LL conditions as independent variables, are reported within the histograms. The 0 value represents straight trajectories; 100 represents perfect circular trajectories; intermediate values represent ovalized trajectories, with the vertical axis longer than the horizontal one. Note, in MN−, the increased OI value in CL respect to LL conditions; in MN+, no modulation of the OI value in the contrast between CL and LL condition. The statistical comparison between the case MN+ and the control MN− is shown; *p < 0.05. Error bars represent standard error of the mean (SEM).

Crawford’s tests revealed that in MN+ patient the discrepancy between the OI values of the two tasks (LL and CL) was significantly smaller than the discrepancies in the healthy subjects (OI value in normative sample [mean ± sd]: LL = 5.5 ± 1.5; CL = 13.4 ± 8.4; corr. between LL and CL = 0.8; in MN+ patient [mean]: LL = 10.4; CL = 11.5; *T* = 4.5; *p* = 0.001, two tailed). No difference between MN− patient and healthy subjects was found (OI value in MN− patient [mean]: LL = 8.0; CL = 32.5; *T* = 0.6; *p* = 0.52, two tailed). Crucial to the present study, directly comparing MN+ and MN− patient, Crawford’s test showed significant differences when considering the OI increase in CL condition with respect to LL condition (difference CL minus LL in MN+ patient [mean]: 1.1; in MN− patient [mean]: 24.4; in normative sample [sd]: 6.8; Z(PCC): −2.4; *p* = 0.03, two tailed). This means that, in CL condition, an OI increase, comparable to that found in healthy subjects, was present only in MN−patient and not in MN+ patient. See Figure 5. With respect to the drawing frequency, Crawford’s test did not show significant difference between MN+ and MN− patients, suggesting that both of them were comparable to the normative sample (Hertz in MN+ patient [mean]: 0.9; in MN− patient [mean]: 1.9; in normative sample [mean ± sd]: 1.3 ± 0.4; Z(PCC): 1.46; *p* = 0.17, two tailed).

### fMRI Results

**Figure 6:**
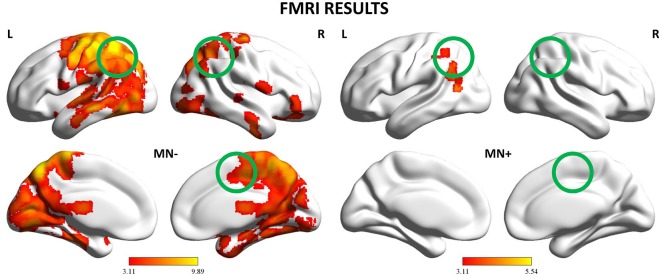
**fMRI results: Whole-brain analysis.** Activation maps for the 2 patients (MN−, MN+) relative to the contrast Bimanual vs. Unimanual actions (CL + LL > L + C). The activation maps are mapped on the lateral and medial views of a MNI template brain at *p* < 0.001 uncorrected. The color bar refers to *t*-values. The position of the tested ROIs are highlighted with circles. MNI coordinates: pre-supplementary motor area (pre-SMA) (4, −2, 52), left posterior parietal cortex (PPC) (−37, −50, 33), right PPC (26, −44, 36).

When contrasting bimanual (LL; CL) against unimanual (L; C) conditions, MN− patient recruited a fronto-parietal network (see Figure 6 and Table 3). In details, in the dominant hemisphere, activation was present within a widespread cluster with peak activity within the left superior parietal lobule, comprising also the left postcentral gyrus and the precuneus. The second cluster was located within the inferior temporal gyrus within the right non-dominant hemisphere and its activation was spreading within the hippocampus. Within the same hemisphere there was also a cluster within the inferior frontal gyrus (pars orbitalis) extending medially within the putamen and the medial prefrontal cortex. Within subcortical structure, there was a bilateral recruitment of the thalamus. Furthermore, activation was also present within other smaller clusters: one within the left precentral gyrus (dorsal premotor cortex) and bilaterally within the inferior frontal gyrus. In details, activation was present in a cluster within the right inferior frontal gyrus, in its most anterior subdivision (pars orbitalis); there was a bilateral recruitment of the pars opercularis of the inferior frontal gyrus, with one cluster located within the left hemisphere and another in the right hemisphere. Within the right hemisphere there was also a cluster in the parietal operculum. Finally, there were two clusters within the temporal cortex: one within the left temporal pole and one within the right superior temporal gyrus.

**Table 3:** **Activation peaks for the contrast between Bimanual and Unimanual actions (CL + LL > L + C) in MN+ and MN− patients**.

Patient MN−					
Peak localization	Voxel number^a^	*x*	*y*	*z*^b^	*t*-value
Left superior parietal lobule	40374	−32	−52	58	9.89
Left postcentral gyrus		−38	−32	58	9.88
Left precuneus		−10	−64	18	9.46
Right inferior temporal gyrus	494	46	−10	−36	6.26
Right inferior temporal gyrus		30	−22	20	5.65
Right hippocampus		54	−10	26	5.41
Right inferior frontal gyrus (Pars orbitalis)	319	24	14	−20	5.78
Right putamen		18	14	−10	4.99
Right medial prefrontal cortex		22	8	−28	4.88
Bilateral thalamus	152	0	−18	12	5.5
Left precentral gyrus	81	−40	2	56	5.43
Right inferior frontal gyrus (Pars orbitalis)	42	48	38	−6	4.58
Left inferior frontal gyrus (Pars opercularis)	42	−52	4	18	4.56
Right inferior frontal gyrus (Pars opercularis)	56	58	4	14	4.05
Right parietal operculum	43	48	−16	24	3.88
Left temporal pole	25	−34	10	−20	3.73
Right superior temporal gyrus	11	58	−26	4	3.35

Patient MN+					

**Peak localization**	**Voxel number^a^**	*x*	*y*	*z*^b^	*t*-value

Left angular gyrus	361	−44	−58	34	5.54
Left angular gyrus		−38	−48	32	4.8
Left angular gyrus		−50	−58	26	4.11
Left middle temporal gyrus	29	−52	−64	14	4.64
Left inferior parietal lobule	21	−28	−48	54	3.66

*^a^For brevity, only clusters with at least 10 contiguous voxels are reported. In red, clusters surviving cluster correction (p < 0.05) are reported. ^b^Stereotaxic coordinates in MNI space are reported in mm*.

By contrast, MN+ patient showed a limited activation pattern comprising mainly one cluster within the left angular gyrus and two smaller clusters one always within the inferior parietal lobule and the other within the middle temporal gyrus (see Figure 6 and Table 3).

#### ROI Analysis: MN− vs. Healthy Participants

Crawford’s test revealed significant difference between MN− patient and healthy subjects in none of the considered ROI (pre-SMA beta value in MN− patient [mean]: LL = 1.64; CL = 1.73; *T* = 2; *p* = 0.08, two tailed; left PPC: LL = 1.07; CL = 1.65; *T* = 0.93; *p* = 0.37, two tailed; right PPC: LL = 0.43; CL = 0.97; *T* = 0.33; *p* = 0.74, two tailed).

#### ROI Analysis: MN+ vs. Healthy Participants

In ROI analysis, Crawford’s tests revealed that in MN+ patient the discrepancy between the beta values for the two tasks (LL and CL) was significantly different with respect to the same discrepancies in healthy subjects for the pre-SMA (beta value in normative sample [mean ± sd]: LL = 0.27 ± 0.28; CL = 0.74 ± 0.53; corr. between LL and CL = 0.87; in MN+ patient [mean]: LL = 0.71; CL = −0.12; *T* = 3; *p* = 0.015, two tailed) and for the left PPC (beta value in normative sample [mean ± sd]: LL = 0.36 ± 0.4; CL = 0.91 ± 0.66; corr. between LL and CL = 0.82; in MN+ patient [mean]: LL = 1.59; CL = 0.91; *T* = 4.14; *p* = 0.002, two tailed); no significant difference for the right PPC was found (beta value in normative sample [mean ± sd]: LL = 0.59 ± 0.34; CL = 1.13 ± 0.54; corr. between LL and CL = 0.83; in MN+ patient [mean]: LL = 0.57; CL = 0.55; *T* = 1.4; *p* = 0.019, two tailed).

#### ROI Analysis: MN+ vs. MN−

**Figure 7:**
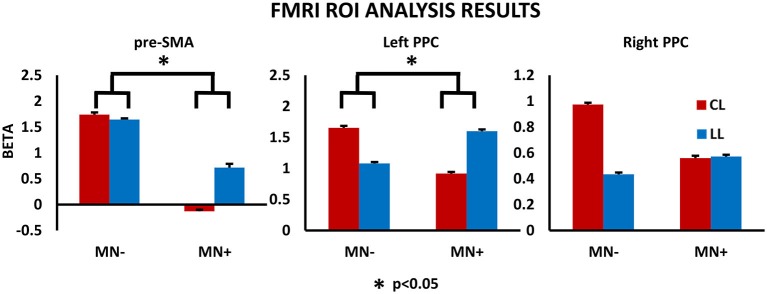
**fMRI results: ROI analysis.** Results for the ROI analysis, in the contrast CL vs. LL, are reported within the histograms for pre-SMA, left PPC and right PPC. MNI coordinates: pre-SMA (4, −2, 52), left PPC (−37, −50, 33), right PPC (26, −44, 36). Note, in MN−, the significant increased beta value in CL respect to LL conditions, for all brain regions; in MN+, the significant decreased beta value in CL respect to LL conditions, for pre-SMA and left PPC, and no modulation between CL and LL condition or right PPC. The statistical comparison between the case MN+ and the control MN− is shown; *p < 0.05. Error bars represent SEM.

Crucial to the present study, directly comparing MN+ and MN− patient, Crawford’s test showed significant differences when considering the beta value increase in CL condition with respect to LL condition, for pre-SMA (difference CL minus LL in MN+ patient [mean]: −0.84; in MN− patient [mean]: 0.09; in normative sample [sd]: 0.32; Z(PCC): 2.35; *p* = 0.04, two tailed) and left PPC (difference CL minus LL in MN+ patient [mean]: −0.67; in MN− patient [mean]: 0.57; in normative sample [sd]: 0.39; Z(PCC): 2.24; *p* = 0.05, two tailed); no significant difference was found for right PPC (difference CL minus LL in MN+ patient [mean]: −0.01; in MN− patient [mean]: 0.53; in normative sample [sd]: 0.32; Z(PCC): 1.19; *p* = 0.26, two tailed). Results for ROI analyses are reported in Figure 7.

## Discussion

In the present case-control study, we investigated the neuro-functional correlates of a behavioral dissociation between congruent and non-congruent bimanual movements in MN syndrome.

The behavioral study showed that, while patients without MN show normal coupling effect in a CL task, MN+ patient did not show any coupling. It is worth noting that the same MN+ patient was tested in a previous behavioral study, employing a similar CL task. At the time of the first test, he was not able to draw left hand circles during the bimanual CL condition and only drew right hand lines. One year later, although in the everyday life the patient spontaneously underused the left hand, he was able to perform bimanual movements, when explicitly required. However, in MN+ patient, a specific impairment in non-congruent bimanual movements was still evident both in ecological action and in the experimental task. Indeed, in LL condition, the MN+ patient could move both hands at the same time, while during CL condition his hands moved asynchronously. Interestingly, according to previous findings on motor awareness in MN syndrome, the patient, when asked to evaluate his performance during the task, was not aware of this specific impairment in CL condition (see Table 2).

In the neuroimaging study, contrasting bimanual (LL; CL) with unimanual (L; C) conditions, MN− patient recruited a fronto-parietal network known to be involved in the execution of bimanual movements (e.g., Nair et al., 2003; Debaere et al., 2004; Wenderoth et al., 2005). Activation was stronger and more widespread within the dominant hemisphere encompassing fronto-parietal networks involved in the planning, execution and online control of hand actions (Filimon, 2010; Turella and Lingnau, 2014; Gallivan and Culham, 2015). On the contrary, MN+ patient showed an abnormal pattern of activity, involving mainly the left angular gyrus even at a rather liberal statistical threshold (*p* < 0.001_uncorr_). This suggests, in MN+ patient, a similar cortical recruitment in both bimanual and unimanual conditions, as if bimanual movements were only a simple sum of unimanual actions. By directly comparing CL and LL conditions, in MN− patient we found that, as previously described in healthy subjects (Garbarini et al., 2013a), a fronto-parietal network, mainly involving pre-SMA and PPC, was significantly more active in non-congruent (CL) than in congruent (LL) conditions. On the contrary, MN+ patient showed an opposite pattern of activation; i.e., in pre-SMA and in left PPC a lower activity in non-congruent (CL) with respect to congruent (LL) conditions.

These behavioral and neuroimaging results are in accordance with a previous demonstration that MN patients fail to inhibit ipsilesional limb motor plans (Coulthard et al., 2008). Using a masked prime task, the authors investigated, in MN patients, the presence of the negative compatibility effect: i.e., the paradoxical reaction time, occurring when the interval between mask and target is 100–200 ms, slower when the prime and target are congruent and faster when they are non-congruent. This study showed that MN patients fail to inhibit the right hand motor plans (evoked by the non-congruent prime), which then intrude abnormally on left hand action planning, slowing down initiation of movement with the left hand. If motor planning for the controlesional arm is intruded by motor plans for the ipsilesional arm, it is likely to expect that congruent bimanual movements will be facilitated and non-congruent bimanual movements will be impaired.

Converging neuroimaging data showed that, during congruent bimanual movements, the (left) non-dominant motor system “entrusts” a part of the control of the non-dominant hand to the (right) dominant motor system via the uncrossed efferent pathway (Aramaki et al., 2006). This normal physiological mechanism, can explain the facilitation in LL condition shown by the MN+ patient, wherein the dominant (intact) motor system implemented the same motor program on both hands. On the contrary, the (right) non-dominant hemisphere has a key role during the execution of bimanual non-congruent movements (Sadato et al., 1997; Wenderoth et al., 2004; Garbarini et al., 2013a). Within this hemispheric balance, the (bilateral) pre-SMA activity is supposed to exert an inhibitory function on the default coupling of homologous muscles, promoted by neural crosstalk, thus allowing the execution of non-congruent bimanual movements (Sadato et al., 1997). The abnormal pre-SMA activity (as well as the related abnormal PPC activity, Wenderoth et al., 2004; Garbarini et al., 2013a) we found in MN+ patient, supports the hypothesis that a failure to inhibit ipsilesional (dominant) motor programs (Coulthard et al., 2008) determines the MN+ patient’s specific impairment in non-congruent CL condition.

From an anatomical point of view, the MN+ patient’s lesion pattern (see Figure 1) was compatible to that described in a recent study (Migliaccio et al., 2014), stressing the role of the cingulum bundle in the MN syndrome. The cingulum is a major pathway of the medial motor system, also connecting this system with limbic structures (e.g., Catani et al., 2013), which underlie motivational aspects of actions (Devinsky et al., 1995). According to Migliaccio et al. (2014), damage to the cingulum is likely to disrupt the integrated functioning of the medial motor system, with subsequent impaired SMA and pre-SMA activity, thus causing the spontaneous underutilization of the contralesional limb. We can speculate that, in the MN+ patient tested here, a partial restoring of this connection between the cingulum and the limbic system, can be the reason of the patient’s behavioral improvement from the first behavioral evaluation (when the patient did not perform bimanual movements in both ecological context or stimulus-driven tasks; see Garbarini et al., 2012) to the present fMRI experiment (when the patient did not spontaneously perform bimanual movements in ecological context, but was able to perform them in stimulus-driven tasks, as the one employed here). Crucially, damage to the cingulum can also lead to an imbalance between left and right medial motor systems, resulting in the specific impaired motor inhibition during non-congruent bimanual movements. Together with the cingulum, it is likely that another fiber bundle can be involved in this lack of inhibition: the SFL I, located just dorsal to the cingulum and, as recently demonstrated in human (Thiebaut de Schotten et al., 2011), connecting the medial parietal and frontal regions, known to play a crucial role in non-congruent bimanual movements. The fiber connections between these areas involved in the task, as well as their possible damage in MN patients, would be a specific matter of interest for future studies.

We acknowledge, as a limitation of the present study, that, being based on only two patients, these results need replication in further studies involving more cases. However, the choice to perform a case-control study was due to the rarity of a pure form of the MN syndrome (without motor deficit), especially in stable patients able to successfully perform a functional task within the MR scanner. Thus, although limited by the sample-size, the present study represent the first neuro-functional investigation of the MN syndrome, showing that an abnormal pre-SMA and parietal activity can lead to a failure to inhibit ipsilesional motor programs, causing both the underutilization of the contralesional limb, characterizing the MN syndrome, and the specific impairment in non-congruent bimanual movements, shown in the present study.

## Funding

This work has been funded by MIUR-PRIN 2012 grant to LF, by Compagnia di San Paolo 2013 grant to AB, by “Futuro in Ricerca” 2013 grant (FIRB 2013, project RBFR132BKP) to LT and by MIUR-SIR 2014 grant (project RBSI146V1D) to FG.

## Conflict of Interest Statement

The authors declare that the research was conducted in the absence of any commercial or financial relationships that could be construed as a potential conflict of interest.

## Footnotes

^1^http://www.mccauslandcenter.sc.edu/mricro/mricron/index.html

^2^SPM5, www.fil.ion.ucl.ac.uk/spm

^3^http://imaging.mrc-cbu.cam.ac.uk/imaging/MniTalairach

## References

[B1] Andersen S. M. Rapcsak S. Z. Beeson P. M. (2010). Cost function masking during normalization of brains with focal lesions: still a necessity? Neuroimage 53, 78–84. 10.1016/j.neuroimage.2010.06.003 20542122 PMC2938189

[B2] Aramaki Y. Honda M. Sadato N. (2006). Suppression of the non-dominant motor cortex during bimanual symmetric finger movement: a functional magnetic resonance imaging study. Neuroscience 141, 2147–2153. 10.1016/j.neuroscience.2006.05.030 16793210

[B3] Ashburner J. Friston K. J. (2005). Unified segmentation. Neuroimage 26, 839–851. 10.1016/j.neuroimage.2005.02.018 15955494

[B4] Buxbaum L. J. Ferraro M. K. Veramonti T. Farne A. Whyte J. Ladavas E.. (2004). Hemispatial neglect: subtypes, neuroanatomy and disability. Neurology 62, 749–756. 10.1212/01.wnl.0000113730.73031.f4 15007125

[B5] Catani M. Dell’acqua F. Thiebaut de Schotten M. (2013). A revised limbic system model for memory, emotion and behaviour. Neurosci. Biobehav. Rev. 37, 1724–1737. 10.1016/j.neubiorev.2013.07.001 23850593

[B6] Classen J. Schnitzler A. Binkofski F. Werhahn K. J. Kim Y. S. Kessler K. R.. (1997). The motor syndrome associated with exaggerated inhibition within the primary motor cortex of patients with hemiparetic. Brain 120, 605–619. 10.1093/brain/120.4.605 9153123

[B7] Cocchini G. Beschin N. Fotopoulou A. Della Sala S. (2010). Explicit and implicit anosognosia or upper limb motor impairment. Neuropsychologia 48, 1489–1494. 10.1016/j.neuropsychologia.2010.01.019 20117119

[B8] Coulthard E. Rudd A. Husain M. (2008). Motor neglect associated with loss of action inhibition. J. Neurol. Neurosurg. Psychiatry 79, 1401–1404. 10.1136/jnnp.2007.140715 19010953 PMC2602747

[B9] Crawford J. R. Garthwaite P. H. Wood L. T. (2010). Inferential methods for comparing two single cases. Cogn. Neuropsychol. 27, 377–400. 10.1080/02643294.2011.559158 21718213

[B10] Crawford J. R. Garthwaite P. H. (2005). Testing for suspected impairments and dissociations in single-case studies in neuropsychology: evaluation of alternatives using monte carlo simulations and revised tests for dissociations. Neuropsychology 19, 318–331. 10.1037/0894-4105.19.3.318 15910118

[B11] Crinion J. Ashburner J. Leff A. Brett M. Price C. Friston K. (2007). Spatial normalization of lesioned brains: performance evaluation and impact on fMRI analyses. Neuroimage 37, 866–875. 10.1016/j.neuroimage.2007.04.065 17616402 PMC3223520

[B12] de Boer B. J. Peper C. L. E. Beek P. J. (2013). Learning a new bimanual coordination pattern: interlimb interactions, attentional focus and transfer. J. Mot. Behav. 45, 65–77. 10.1080/00222895.2012.744955 23406196

[B13] Debaere F. Wenderoth N. Sunaert S. Van Hecke P. Swinnen S. P. (2004). Cerebellar and premotor function in bimanual coordination: parametric neural responses to spatiotemporal complexity and cycling frequency. Neuroimage 21, 1416–1427. 10.1016/j.neuroimage.2003.12.011 15050567

[B14] Devinsky O. Morrell M. J. Vogt B. A. (1995). Contributions of anterior cingulate cortex to behaviour. Brain 118, 279–306. 10.1093/brain/118.1.279 7895011

[B15] Dounskaia N. Nogueira K. G. Swinnen S. P. Drummond E. (2010). Limitations on coupling of bimanual movements caused by arm dominance: when the muscle homology principle fails. J. Neurophysiol. 103, 2027–2038. 10.1152/jn.00778.2009 20071629 PMC2853291

[B16] Filimon F. (2010). Human cortical control of hand movements: parietofrontal networks for reaching, grasping and pointing. Neuroscientist 16, 388–407. 10.1177/1073858410375468 20817917

[B17] Franz E. A. Ramachandran V. S. (1998). Bimanual coupling in amputees with phantom limbs. Nat. Neurosci. 1, 443–443. 10.1038/2161 10196540

[B18] Franz E. A. Zelaznik H. N. McCabe G. (1991). Spatial topological constraints in a bimanual task. Acta Psychol. (Amst) 77, 137–151. 10.1016/0001-6918(91)90028-x 1759589

[B19] Gallivan J. P. Culham J. C. (2015). Neural coding within human brain areas involved in actions. Curr. Opin. Neurobiol. 33, 141–149. 10.1016/j.conb.2015.03.012 25876179

[B20] Garbarini F. Pia L. (2013). Bimanual coupling paradigm as an effective tool to investigate productive behaviors in motor and body awareness impairments. Front. Hum. Neurosci. 7:737. 10.3389/fnhum.2013.00737 24204339 PMC3817803

[B21] Garbarini F. D’Agata F. Piedimonte A. Sacco K. Rabuffetti M. Tam F.. (2013a). Drawing lines while imagining circles: neural basis of the bimanual coupling effect during motor execution and motor imagery. Neuroimage 88, 100–112. 10.1016/j.neuroimage.2013.10.061 24188808

[B22] Garbarini F. Pia L. Piedimonte A. Rabuffetti M. Gindri P. Berti A. (2013b). Embodiment of an alien hand interferes with intact-hand movements. Curr. Biol. 23, R57–R58. 10.1016/j.cub.2012.12.003 23347936

[B23] Garbarini F. Piedimonte A. Dotta M. Pia L. Berti A. (2013c). Dissociations and similarities in motor intention and motor awareness: the case of anosognosia for hemiplegia and motor neglect. J. Neurol. Neurosurg. Psychiatry 84, 416–419. 10.1136/jnnp-2012-302838 22955177

[B24] Garbarini F. Rabufetti M. Piedimonte A. Solito G. Berti A. (2015). Bimanual coupling effects during arm immobilization and passive movements. Hum. Mov. Sci. 41, 114–126. 10.1016/j.humov.2015.03.003 25797919

[B25] Garbarini F. Rabuffetti M. Piedimonte A. Pia L. Ferrarin M. Frassinetti F.. (2012). “Moving” a paralysed hand: bimanual coupling effect in patients with anosognosia for hemiplegia. Brain 135, 1486–1497. 10.1093/brain/aws015 22374937

[B26] Gauthier L. Dehaut F. Joanette Y. (1989). The bells test: a quantitative and qualitative test for visual neglect. Int. J. Clin. Neuropsychol. 11, 49–54.

[B27] Gold M. Adair J. C. Jacobs D. H. Heilman K. M. (1994). Anosognosia for hemiplegia: an electrophysiologic investigation of the feed-forward hypothesis. Neurology 44, 1804–1808. 10.1212/wnl.44.10.1804 7936225

[B28] Laplane D. Degos J. D. (1983). Motor neglect. J. Neurol. Neurosurg. Psychiatry 46, 152–158. 10.1136/jnnp.46.2.152 6842219 PMC1027298

[B29] Measso G. Cavarzeran F. Zappala G. Lebowitz B. D. Crook T. H. Pirozzolo F. J. (1993). The mini-mental state examination: normative study of an italian random sample. Dev. Neuropsychol. 9, 77–85. 10.1080/87565649109540545

[B30] Migliaccio R. Bouhali F. Rastelli F. Ferrieux S. Arbizu C. Vincent S.. (2014). Damage to the medial motor system in stroke patients with motor neglect. Front. Hum. Neurosci. 8:408. 10.3389/fnhum.2014.00408 24966826 PMC4052665

[B31] Nair D. G. Purcott K. L. Fuchs A. Steinberg F. Kelso J. A. (2003). Cortical and cerebellar activity of the human brain during imagined and executed unimanual and bimanual action sequences: a functional MRI study. Brain Res. Cogn. Brain Res. 15, 250–260. 10.1016/s0926-6410(02)00197-0 12527099

[B32] Pia L. Spinazzola L. Garbarini F. Bellan G. Piedimonte A. Fossataro C.. (2014). Anosognosia for hemianaesthesia: a voxel-based lesion-symptom mapping study. Cortex 61, 158–166. 10.1016/j.cortex.2014.08.006 25481473

[B33] Pia L. Spinazzola L. Rabuffetti M. Ferrarin M. Garbarini F. Piedimonte A.. (2013). Temporal coupling due to illusory movements in bimanual actions: evidence from anosognosia for hemiplegia. Cortex 49, 1694–1703. 10.1016/j.cortex.2012.08.017 23021071

[B34] Ridderikhoff A. Daffertshofer A. Peper C. E. Beek P. J. (2005). Mirrored EMG activity during unimanual rhythmic movements. Neurosci. Lett. 381, 228–233. 10.1016/j.neulet.2005.02.041 15962399

[B35] Sadato N. Yonekura Y. Waki A. Yamada H. Ishii Y. (1997). Role of the supplementary motor area and the right premotor cortex in the coordination of bimanual finger movements. J. Neurosci. 17, 9667–9674. 9391021 10.1523/JNEUROSCI.17-24-09667.1997 PMC6573404

[B36] Saevarsson S. (2013). Motor response deficits of unilateral neglect: assessment, therapy and neuroanatomy. Appl. Neuropsychol. Adult 20, 292–305. 10.1080/09084282.2012.710682 23590244

[B37] Siekierka-Kleiser E. M. Kleiser R. Wohlschlager A. M. Freund H. J. Seitz R. J. (2006). Quantitative assessment of recovery from motor hemineglect in acute stroke patients. Cerebrovasc. Dis. 21, 307–314. 10.1159/000091535 16490939

[B38] Spencer R. M. C. Ivry R. B. Cattaert D. Semjen A. (2005). Bimanual coordination during rhythmic movements in the absence of somatosensory feedback. J. Neurophysiol. 94, 2901–2910. 10.1152/jn.00363.2005 16014794

[B39] Swinnen S. P. Puttemans V. Vangheluwe S. Wenderoth N. Levin O. Dounskaia N. (2003). Directional interference during bimanual coordination: is interlimb coupling mediated by afferent or efferent processes. Behav. Brain Res. 139, 177–195. 10.1016/s0166-4328(02)00266-8 12642188

[B40] Tam F. Churchill N. W. Strother S. C. Graham S. J. (2012). A new tablet for writing and drawing during functional MRI. Hum. Brain Mapp. 32, 240–248. 10.1002/hbm.21013 20336688 PMC6870006

[B41] Thiebaut de Schotten M. Dell’acqua F. Forkel S. J. Simmons A. Vergani F. Murphy D. G.. (2011). A lateralized brain network for visuospatial attention. Nat. Neurosci. 14, 1245–1246. 10.1038/nn.2905 21926985

[B42] Turella L. Lingnau A. (2014). Neural correlates of grasping. Front. Hum. Neurosci. 8:686. 10.3389/fnhum.2014.00686 25249960 PMC4158794

[B43] Wenderoth N. Debaere F. Sunaert S. Swinnen S. (2005). The role of anterior cingulate cortex and precuneus in the coordination of motor behaviour. Eur. J. Neurosci. 22, 235–246. 10.1111/j.1460-9568.2005.04176.x 16029213

[B44] Wenderoth N. Debaere F. Sunaert S. van Hecke P. Swinnen S. P. (2004). Parieto-premotor areas mediate directional interference during bimanual movements. Cereb. Cortex 14, 1153–1163. 10.1093/cercor/bhh075 15142955

